# Healthcare Professionals’ Experiences of Barriers and Facilitators to ARFID Identification, Diagnosis and Treatment: A Rapid Review

**DOI:** 10.1002/erv.70148

**Published:** 2026-07-11

**Authors:** Sandra‐Eve Bamigbade, Freya Cooper, Amanda Ludlow

**Affiliations:** ^1^ Department of Psychology and Sport Science School of Life and Medical Sciences University of Hertfordshire Hatfield UK; ^2^ Complex Feeding Team, Department of Paediatric Neurosciences Evelina London Children’s Hospital Guy’s and St Thomas’ NHS Foundation Trust London UK

**Keywords:** avoidant/restrictive food intake disorder (ARFID), barriers and facilitators, diagnosis and treatment, healthcare professionals, rapid review

## Abstract

**Objectives:**

Despite increasing evidence of the physical and psychosocial impairments associated with Avoidant/Restrictive Food Intake Disorder (ARFID), there are currently no national guidelines for its assessment and treatment. In the absence of such guidance, it is crucial to understand the experiences of healthcare professionals (HCPs) involved in ARFID care.

**Method:**

This review included 13 studies exploring HCPs’ experiences of accessing or delivering care for individuals with ARFID. The findings were synthesised into three key themes (Kambanis and Thomas 2023): limited knowledge surrounding ARFID (American Psychiatric Association 2022), lack of education and specialised training and (World Health Organization 2019) the need for clear referral pathways and multidisciplinary collaboration.

**Results:**

The review identified significant gaps in HCPs’ understanding of ARFID, including a lack of confidence in clinical decision‐making, challenges with differential diagnosis, difficulties navigating referral processes and an absence of established care pathways. These barriers contribute to inconsistent care provision across services.

**Conclusions:**

Addressing these gaps through enhanced training, defined referral pathways and improved multidisciplinary collaboration is essential for improving diagnostic accuracy and treatment outcomes. While this review may represent a snapshot of HCP awareness during a period of growing recognition of ARFID, the themes identified appear consistent across settings. These findings underscore the need for a global effort to build HCPs' confidence and competence in recognising and managing ARFID, thereby improving patient outcomes.

## Highlights

- Healthcare professionals (HCPs) often reported limited confidence in supporting individuals with ARFID, reflecting gaps in training and awareness, particularly in relation to co‐occurring neurodevelopmental conditions.
- Systemic barriers to referral, such as restrictive exclusion criteria, inconsistent thresholds and the absence of established care pathways, contribute to fragmented and delayed access to care.
- Viewing ARFID solely through an eating disorder framework may restrict access to appropriate support. Multidisciplinary collaboration is essential to address the overlap with neurodevelopmental and feeding‐related difficulties.

## 1 Introduction

Avoidant Restrictive Food Intake Disorder (ARFID) is a clinically significant eating disorder characterised by avoidance of certain foods or food groups and/or restricted overall food intake, resulting in marked nutritional, weight‐related and/or psychosocial impairment. ARFID may involve difficulties maintaining adequate weight, nutritional deficiencies (or reliance on supplements) and impaired psychosocial functioning, and occurs in the absence of concerns about body weight or shape (Kambanis and Thomas 2023). It was first introduced in the fifth edition of the Diagnostic and Statistical Manual of Mental Disorders (DSM‐5) (American Psychiatric Association 2022) and the 11th revision of the International Classification of Diseases (ICD‐11) (World Health Organization 2019). However, despite increasing recognition of ARFID, the provision of care continues to vary significantly (Willmott et al. 2024), and established care pathways remain limited or inconsistent across services (Duffy et al. 2024).

One of the complexities of ARFID lies in its heterogeneous clinical presentation, leading to little consensus on its prevalence. A systematic review by Bourne et al. 2020 reported estimates ranging widely from 1.5% to 64.0%, likely reflecting differences in study populations, diagnostic criteria or assessment methods. While ARFID can manifest at any age, it is most commonly diagnosed in children and adolescents (Katzman et al. 2021). Research has debated the existence of sex differences, with some studies suggesting a higher prevalence among males (Katzman et al. 2019), while others report no notable differences (Watts et al. 2023).

The relationship between ARFID and weight status is also not clear. For example, those with fear or lack of interest as a driver tend to lean towards being underweight and report greater weight loss (Sanchez‐Cerezo et al. 2024). In contrast, individuals who have increased sensory sensitivities as a driver, vary more greatly across the weight spectrum (Zickgraf et al. 2019). This diversity in weight status underscores the importance of not assuming a uniform presentation of ARFID based on weight alone and highlights the need for careful assessment of an individual’s specific drivers and nutritional needs.

Among the ARFID population, co‐occurring psychiatric conditions are also highly prevalent, with many meeting the criteria for more than one psychiatric disorder, with estimates ranging from 10% to 25% (Cooney et al. 2018; Lieberman et al. 2019), including anxiety disorders with prevalence estimates ranging from 9.1% to 72% (Sanchez‐Cerezo et al. 2023), mood disorders ranging 17%–33% (Duncombe Lowe et al. 2019) and Autism ranging 9.2%–54.75% (Sanchez‐Cerezo et al. 2023). This highlights the importance of considering the occurrence of other psychiatric conditions when treatment planning for ARFID.

The heterogeneity in ARFID presentation means that individuals requiring treatment are often seen by multiple healthcare professionals (HCPs) (LaMarre et al. 2023). For example, in the UK, a GP might make the initial referral, children with ARFID could be treated by their local Child and Adolescent Mental Health Services (CAMHS), community eating disorder CAMHS, community and acute paediatric services or by a range of practitioners, including dieticians, speech and language therapists and other specialists. In contrast, adults with ARFID may be treated by specialist eating disorders services, general mental health services or by different practitioners. Despite the involvement of diverse healthcare professionals, little is known about the barriers and facilitators they encounter when delivering care to individuals with ARFID.

### 1.1 Aim

This review aimed to identify and synthesise HCPs experiences of the identification, diagnosis and treatment of ARFID, with particular reference to their knowledge, attitudes, beliefs and clinical experiences.

### 1.2 Hypothesis

Given the lack of standardised clinical guidelines for ARFID, it was hypothesised that HCPs may encounter challenges in the identification, diagnosis and treatment of the disorder, potentially resulting in variations in care provision across disciplines, and services.

## 2 Materials and Methods

This review was undertaken following the Preferred Reporting Items for Systematic Reviews and Meta‐Analysis (PRISMA) guidelines (Page et al. 2021) a literature search was conducted. The review protocol was registered (CRD42023447759) 24/10/2023.

### 2.1 Inclusion Criteria

Peer‐reviewed articles and published papers employing all methodologies were screened for inclusion. Studies were eligible if they focused on the experiences, attitudes and perceptions of HCPs in the delivery of healthcare for individuals with ARFID internationally. Although literature reviews were excluded from the analysis, their reference lists were examined to identify additional relevant studies for inclusion.

### 2.2 Population

This review aimed to comprehensively explore the published literature on HCPs accounts of providing care and support for ARFID. Studies were eligible for inclusion if they involved HCPs who had directly worked with individuals diagnosed with ARFID.

### 2.3 Context

All stages in the healthcare pathway were included, for example, primary, secondary and tertiary, and papers were not limited by date of publication or geographical location.

### 2.4 Exclusion Criteria

Literature reviews were not included, but their reference lists were hand searched for additional papers. Case studies, thesis/dissertations, opinion articles, grey literature and any papers that did not specifically discuss the experiences of health professionals working ARFID were also excluded. All papers had to be available in English Language. Grey literature was excluded to prioritise peer‐reviewed evidence and the synthesis of established clinical perspectives relating to ARFID, while maintaining a manageable and methodologically consistent review process.

### 2.5 Search Strategy

PROSPERO was initially searched to identify any pre‐existing systematic reviews on the topic. Following this, the databases PubMed, PsycARTICLES, CINAHL Plus and Scopus were systematically searched for relevant studies. In accordance with guidance from Siddaway et al. 2019, the search string was developed based on key concepts and incorporated various terminologies representing equivalent constructs (see Table 1).

**TABLE 1:** Search terms.

Condition	Professional	Outcome
1. Avoidant/Restrictive food intake disorder 2. ARFID 3. Selective eating disorder	1. Clinician* 2. Healthcare provider* 3. Healthcare professional* 4. Therapist* 5. Psychiatrist* 6. Psychologist* 7. Paediatrician* 8. Physician*	1. Barrier* 2. Facilitator* 3. Challenge* 4. Obstacle* 5. Enabler* 6. Perspective* 7. Experience*

*Note:* The asterisk (*) was used as a truncation wild card to retrieve variations of a word stem.

The search strategy combined terms related to the condition (ARFID), professional roles (HCPs) and outcomes of interest (experiences and related factors). The following search logic was applied.

Condition terms AND Professional terms

OR

Condition terms AND Outcome terms

A decision was made to exclude older or non‐psychiatric feeding terms (e.g., Paediatric Feeding Disorder) to maintain focus on ARFID as conceptualised within contemporary mental health and eating disorder frameworks, rather than encompassing a broader range of feeding difficulties defined within alternative diagnostic systems. This approach was intended to preserve diagnostic specificity and reduce the risk of conceptual overlap or misclassification. Selective Eating Disorder was retained as a search term because it has been identified within the literature as a direct antecedent construct to ARFID, with substantial phenomenological overlap.

### 2.6 Screening Process

The initial search conducted on 2 March 2024 yielded 778 records. Utilising Covidence software, duplicates were identified and removed, resulting in 710 unique citations for screening. An updated search in March 2025 did not yield any new studies eligible for inclusion. A further review was carried out in May 2026, and this provided a further four articles to be considered.

The first author conducted the initial screening of all titles and abstracts against the predefined inclusion and exclusion criteria. To ensure consistency and accuracy, the third author (AL) independently screened 10% of the articles using the same criteria.

Following this, 120 records underwent full‐text review. Both reviewers screened these records independently to assess eligibility and reach consensus on inclusion.

### 2.7 Data Extraction

To capture the diversity of the included studies, a thematic synthesis approach was employed. There were 11 studies that met the inclusion criteria from the original searches and 4 from the latest search. Data were extractable from 13 and included in the synthesis. The data were analysed to identify overarching themes and sub‐themes related to HCPs’ knowledge of ARFID and their experiences managing the disorder, with particular emphasis on barriers and facilitators encountered across professional tasks. Thematic analysis combined deductive and inductive approaches, using line‐by‐line coding to explore predefined themes while remaining open to novel insights.

### 2.8 Assessment of Methodological Quality

All studies that met the inclusion criteria were critically appraised using Joanna Briggs Institute (JBI) Critical Appraisal Checklists (Barker et al. 2024).

### 2.9 Data Synthesis

The data were synthesised via a narrative synthesis approach, elucidating commonalities, differences and observed patterns across the included studies.

## 3 Results

In addition to the international study, this review included research from different eight countries and included 5970 HCPs, see Table 2. The largest participant groups included studies were mental health and primary care professionals (*n* = 2288), followed by Yogo teachers (*n* = 1886). Yogo teachers may be classified as healthcare professionals based on the function they perform. In Japan, Yogo teachers replace school nurses and are licenced educations that support the growth and development of children through health and education services (Okada 2011). Together, mental health and primary care professionals and Yogo teachers constituted the majority of the HCPs represented in this review. Regarding ARFID populations represented across studies, nine studies included HCPs who work within children and adolescents, two included HCPs working across the lifespan, and two did not specify the population focus, though these likely also included HCPs working across the lifespan.

**TABLE 2:** Characteristics of studies included in the review.

Year first author	Country	Design/Method	Study aims	HCP type (*n*,%) and ARFID population
2014 Katzman	Canada	Cross‐sectional Survey	Not named (case report including unpublished data	**HCP type:** Paediatricians and paediatric subspecialists (*n* = 657) **ARFID population:** Paediatric
2016 Seike^a^	Japan	Cross‐sectional Survey	To calculate the encounter rate by ED type (based on DSM‐5), and examine the relations between the rates and location, school type, number of students, years of experience as a Yogo teacher, nursing experience and eating disorder knowledge.	**HCP type:** Yogo Teacher^c^ (*n* = 1886)**ARFID population:** Paediatric
2018 Guss^a^	USA	Mixed‐method survey	To determine the current protocols and practices used for inpatient medical stabilisation of patients with ARFID in the United States.	**HCP type**: Physicians (*n* = 37)**ARFID population:** Paediatric
2019 Claudino	International	Experimental vignette‐based case‐control online survey	To assess the clinical utility of the ICD‐11 diagnostic guidelines for ARFID, including their clarity, ease of use, reliability and applicability across diverse clinical settings.	**HCP type:** Mental health and primary care professionals (*n* = 2288) from the following disciplines: Medicine (*n* = 1367, 59.7%) Psychology (*n* = 693, 30.3%) Counselling (*n* = 85, 3.7%) Nursing (*n* = 49, 2.1%) Social work (*n* = 24, 1.0%) Sex therapy (*n* = 6, 0.3%) Speech therapy (*n* = 2, 0.1%) Other (*n* = 62, 2.7%) **ARFID population: Mixed**
2021 Magel	Canada	Qualitative (presented in letter to the editor) Semi‐structured interviews	Individuals with ARFID often seek help from various HCPs, many of whom do not specialise in mental health treatment and may be unfamiliar with ARFID. This study sought to fill this knowledge gap and evidence the need for training among community clinicians.	**HCP type:** Clinicians (*n* = 35) from the following disciplines:Mental health professionals (*n* = 20, 56.5%) Occupational therapists (*n* = 8, 21.7%) Dietitians (*n* = 6, 17.4%) Physicians (*n* = 1, 4.4%) **ARFID population:** Unspecified
2021 Coelho	Canada	Cross sectional with vignettes Online survey	Two aims: 1. To elucidate health professionals’ familiarity and experience in working with ARFID in a broad sample of multi‐disciplinary paediatric health professionals. 2. To assess the ability of health professionals with experience in working with ARFID to differentiate between different clinical presentations of restrictive eating.	**HCP type:** Health professionals (*n* = 93) from the following disciplines: Dietitians (*n* = 25, 26.9%) Paediatrician/adolescent health physicians (*n* = 18, 19.3%) Nurse/psychiatric nurses (*n* = 10, 10.8%) Medical/paediatric residents (*n* = 9, 9.7%) Primary care provider/family physicians (*n* = 8, 8.6%) Psychologists (*n* = 8, 8.6%) Social workers (*n* = 5, 5.4%) Therapist/clinical supports (i.e., registered clinical counsellor, child and youth worker, *n* = 5, 5.4%) Other (i.e., psychiatrist, internist; occupational therapist, speech‐language pathologist, *n* = 5, 5.4%) **ARFID population:** Paediatric
2021 Harrison^a^	UK	Mixed method Online survey	Two aims: 1. To investigate the level of HCP confidence in identifying and referring paediatric patients with ARFID, focussing on HCPs working in both primary and secondary (specialist) care in the UK. 2. To report and describes HCPs experiences of any current barriers to effective healthcare for children with ARFID.	**HCP type**: Primary and secondary care professionals (*n* = 45) from the following disciplines: Health visitors (*n* = 14, 31.1%) Paediatricians (*n* = 8, 17.8%) Speech and language therapists (*n* = 8, 17.8%) Psychologists (*n* = 7, 15.6%) Dieticians (*n* = 4, 8.9%) General practitioners (*n* = 3, 6.7%) School nurse (*n* = 1, 2.2%) **ARFID population:** Paediatric
2022 Jackson^b^	New Zealand	Mixed method with Vignette Online survey	To explore health professionals’ understanding of children with picky eating and the consensus among professions for the labelling of a particular condition portrayed in a vignette.	**HCP type**: Health professionals (*n* = 73 in 2013, 68 in 2018) from the following disciplines: Medical practitioners 2013 (*n* = 13, 17.8%) 2018 (*n* = 12, 17.7%) Dietitians 2013 (*n* = 15, 20.6%) 2018 (*n* = 18, 26.5%) Speech‐language therapists 2013 (*n* = 45, 61.6%) 2018 (*n* = 29, 42.7%) Other 2013 (*n* = 0) 2018 (*n* = 9, 13.2%) **ARFID population:** Paediatric
2022 Raffoul	USA	Quasi experimental Online survey	Pilot‐test the efficacy of online training to improve paediatric HCPs comfort in screening and referrals, knowledge about eating disorders and behaviours related to screening for eating disorders and referral to services.	**HCP type:** Paediatrics primary care (*n* = 84) from the following disciplines: Physicians (*n* = 21, 25.3%) Nurses (*n* = 51, 60.2%) Other or did not respond (*n* = 12, 14.5%) **ARFID population:** Paediatric
2023 Dinkler	Sweden	Cross sectional Mixed methods online survey	Assess self‐reported knowledge and confidence regarding ARFID diagnosis and treatment in Swedish HCPs from various disciplines	**HCP type:** Clinicians (*n* = 489) from the following disciplines: Speech and language therapist (*n* = 138, 28.2%) dietician (*n* = 60, 12.3%) nurse (*n* = 58, 11.9%) psychologist (*n* = 56, 11.4%) counsellor (*n* = 37, 7.6%) physician (*n* = 12, 2.5%) occupational therapist (*n* = 10, 2.0%) special education teacher (*n* = 7, 1.4%) other (*n* = 49, 10.0%) **ARFID population**: Mixed
2025 Trancón	Spain	Cross‐sectional Online survey	To analyse terminology and professionals’ views on features and indicators related to ARFID among Spanish professionals working with autistic individuals, identifying potential gaps and training needs.	**HCP type**: Clinicians (*n* = 194) from the following disciplines: Speech therapists (*n* = 62, 31.9%) Psychologists (*n* = 62, 31.9%) Occupational therapist (*n* = 70, 36%) **ARFID population**: Unspecified
2025 Harrison	UK	Cross‐sectional A mixed methods online survey	To investigate and categorises the different barriers to effective healthcare for children with ARFID, from the perspective of those who encounter and care for these children in their professional practice	**HCP type**: Clinicians (*n* = 45) from the following disciplines: Health visitor (*n* = 14; 31.1%) Paediatrician (*n* = 8; 17.8%) Speech and language therapist (*n* = 8; 17.8%) Psychologist (*n* = 7, 15.6%) Dietitian (*n* = 4, 8.9%) GP (*n* = 3, 6.7%) School nurse (*n* = 1, 1 2.2%) **ARFID population**: Paediatric
2026 Ford	Ireland	Qualitative Semi‐structured interviews	To explore the perspectives of healthcare professionals on the impact of COVID‐19 on EDs and ED treatment and service provision for the paediatric population	**HCP type**: Clinicians (*n* = 13) from the following disciplines: Psychotherapist (*n* = 4, 30.7%) Clinical psychologist (*n* = 3, 23%) Clinical nurse & education facilitator (*n* = 1, 7.7%) Eating disorder Specialist/nurse practitioner (*n* = 2) Nutritionist (*n* = 1, 7.7%) Educational psychologist and peer reviewed ACT trainer (*n* = 1, 7.7%) psychologist, counsellor and psychotherapist (*n* = 1, 7.7%) **ARFID population**: Paediatric

^a^ Studies located through snowballing technique.

^b^ Study located during full‐text review screening as referenced in a study and appeared to meet criteria for this review.

^c^ Yogo teachers are unique teachers in Japan that act as school nurses as part of their role. They are health educators responsible for weighing students and reviewing medical records to screen for medical concerns.

### 3.1 Limited Knowledge Surrounding ARFID

#### 3.1.1 Lack of Certainty Over Symptoms of ARFID

Awareness of ARFID was found to be limited across different HCPs. For example, Katzman et al. 2014 found that 63% of Paediatricians and Paediatric subspecialists were unfamiliar with ARFID. Magel et al. 2021 also revealed that half (51.4%) of HCPs, predominantly Mental Health Professionals, had not heard of ARFID previously, despite two‐thirds (65.7%) encountering patients with ARFID presentations.

Harrison 2021 captured similar uncertainty among UK HCPs, particularly Health Visitors, who expressed reservations about recognising and confidently diagnosing ARFID. Whilst many primary care HCPs (67%, e.g., GPs, practice nurses and pharmacists) did not agree that they could identify a child with ARFID, most of the secondary care HCPs (74%, i.e., specialist/community services) felt they would be able to. Notably, Coelho et al. 2021 also found primary care clinicians reporting low confidence compared to specialists, with 78.5% of HCPs, predominantly Dieticians, reported familiarity with the diagnosis, which was found to be higher in those who provided care in their clinical practice for paediatric feeding or eating disorders. Although more recent evidence suggests these difficulties persist across both groups despite varying levels of familiarity (Harisson 2025).

Similarly, 86.6% of Swedish HCPs expressed a lack of confidence in their ability to diagnose ARFID (Coelho et al. 2021). They found HCPs working in Child and Adolescent Habilitation Units exhibited the highest proportion of professionals (95.4%) reporting a lack of confidence in their ability to diagnose ARFID, compared to those HCPs predominantly working with eating disorders (82%) and Physicians and Psychologists (68.2%).

Dinkler et al. 2023 also explored whether HCPs were familiar with the diagnostic tools available to assess ARFID; only 8.6% (12.3% among Physicians/Psychologists) reported knowing which tools they could use to assess and diagnose ARFID. Concerns were raised about the insufficient familiarity with these tools, particularly among Physicians and Psychologists, who play a pivotal role in assessing and diagnosing ARFID. This was further reiterated by Magel et al. 2021. In Japan, where Yogo teachers often oversee the daily health of the whole school, including eating concerns, Seike et al. 2016 found the teachers prioritised the need for a list of medical/consultation institutions to support them further.

#### 3.1.2 Differentiating ARFID From Selective Eating, Autism and Anorexia

Harrison 2021; Harisson 2025 have noted the general lack of ARFID awareness among British HCPs, particularly when distinguishing it from ‘fussy eating’. Moreover, Dinkler et al. 2023 found the majority of Swedish HCPs (70%–90%) indicated difficulty distinguishing ARFID from Autism and selective eating. Specifically, less than a third (28.3%) felt able to discern ARFID from Autism, and even fewer (9.7%) felt adept at differentiating ARFID from selective eating. A finding reiterated by Spanish professionals Tracón and López‐Resa 2025. Magel et al. 2021 also discussed challenges differentiating ARFID from Autism, suggesting that ARFID symptoms might be ‘overshadowed’ by Autistic symptoms due to a shared presentation of ridged, obsessive or repetitive behaviours and sensory sensitivities. However, this assumption implies that ARFID and Autism are mutually exclusive disorders, when they often coexist (Farag et al. 2022).

Patterns of occurrence of eating disorders were believed to have changed over time, with qualitative interviews of HCPs in Ireland, highlighting the impact of COVID‐19 on referrals (Ford and Carr 2026). HCPs believed that children with ARFID were more likely to present with additional conditions like anxiety, OCD and Autism made the eating disorders harder to recognise and diagnose.

Recognising ARFID extends beyond a superficial understanding of the condition. Harrison 2021 illustrated this well through their use of clinical vignettes to assess HCPs’ ability to correctly identify different restrictive eating presentations. While HCPs were skilled at differentiating restrictive eating without an ARFID diagnosis, those with psychosocial impairment alone, and those with restrictive eating and excessive exercise (anorexia), were less accurately classified. Further analysis revealed there was ambiguity over whether ARFID must be associated with weight loss or failure to gain weight, with ARFID being correctly identified in 76.7% of HCPs who did not believe in this criterion compared with 48.4% of those who did. This discrepancy suggests a potential cognitive dissonance among those who endorsed the criterion yet still identified ARFID. For instance, Jackson et al. 2022 found while most HCPs agreed on reduced food quantity and variety as defining features of ‘picky eating’, they diverged on causal factors, resultant behaviours and the impacts. Speech and Language Therapists focused on sensory influences and family mealtimes, whilst Dieticians emphasised the chronicity of food refusal and gave examples of specific mealtime behaviours such as food spitting and crying.

### 3.2 Need for Education and Specialised Training

#### 3.2.1 Lack of ARFID‐Training, Education and Resources

Dinkler et al. 2023 revealed a majority of Swedish HCPs (64%) reported never having received formal ARFID training, with only 4.3% of the sample considered themselves as ARFID experts. Additionally, 25.8% received less than 2 h of training, 7.9% had less than 10 h and only 2.3% of HCPs received at least 10 h of training, highlighting a substantial gap in knowledge acquisition and depth of learning. Further analysis revealed that education levels and subsequent knowledge varied among different types of HCPs. For example, Physicians/Psychologists and Eating Disorder Clinicians exhibited higher levels of knowledge compared to Neurodevelopmental and Child and Adolescent Habilitation Clinicians. This discrepancy may be attributed to role‐specific continuous professional development and increased exposure within specific clinical settings, necessitating further training.

Other studies highlighted similar challenges with lack of resources and training in the UK and New Zealand (Harrison 2021; Jackson et al. 2022). Additionally, only 8.6% of Canadian HCPs treating patients with ARFID reported having received any ARFID‐related training (Magel et al. 2021). This suggests that a significant majority feel they are providing care beyond the scope of their clinical training. Raffoul et al. 2022 conducted a pilot study to assess the effectiveness of an online training video aimed at enhancing the comfort, knowledge and screening behaviours of US Paediatric HCPs regarding eating disorders, including ARFID. Prior to the webinar, 40.85% of HCPs reported feeling ‘*not at all comfortable*’ with screening for ARFID, to 1.43% following the webinar and 0% at follow‐up training. Additionally, significant enhancements were observed in HCPs' knowledge particularly in correctly identifying tools for screening ARFID (38.36% at baseline, 54.22% post‐video).

#### 3.2.2 Lack of Familiarity With Diagnostic Criteria

Notably, Magel et al. 2021 found that half of their Canadian HCPs were unfamiliar with the diagnostic criteria. This was reaffirmed by Coelho et al. 2021 who found a significant proportion of Canadian HCPs (78.3%) recognised ARFID as involving a persistent failure to meet nutritional or energy needs, while 50% believed it must be associated with substantial weight loss or failure to achieve expected weight gain, or faltering growth. Only a third (37%) could correctly identify all the DSM‐5 diagnostic criteria, with those experienced in ARFID showing higher proficiency identifying all criteria (47.5%) compared to those who were inexperienced (16.1%). In Spain, a survey of 194 professionals including occupational therapists, psychologists and speech and language therapists revealed that the majority (61%), reported uncertainty around ARFID diagnostic criteria, and just 34% were familiar with validated assessment tools (Tracón and López‐Resa 2025). While the psychologist were found to report greater familiarity with DSM‐5 criteria, speech and occupational therapists tended to emphasise sensory and behavioural features.

Akin to Coelho et al. 2021, Jackson et al. 2022 also used a clinical vignette to gauge how HCPs would classify a case and whether there were any changes from 2013 to 2018. They presented the case of a 3‐year‐old, providing pertinent information supporting a potential ARFID diagnosis. HCPs, predominantly Speech and Language Therapists, were asked whether they believed there was a consensus in the medical field for labelling this condition, revealing a significant shift in opinion between 2013 and 2018, though not as expected. In 2013, a majority (75.3%) of HCPs agreed there was no consensus on a label for the vignette. Surprisingly, this increased to 89.7% in 2018 despite the introduction of ARFID into the DSM‐5. Further exploration uncovered a notable decrease in ‘I don’t know’ responses (15.1% in 2013, 2.9% in 2018), and an increase in those indicating ‘no consensus’ (75.3% in 2013, 89.7% in 2018). Furthermore, according to Katzman et al. 2014 unpublished findings from the Canadian Paediatric Surveillance Programme, Paediatricians and Paediatric subspecialists retrospectively reported encountering 339 cases of ARFID within the past year. Surprisingly, a third (30%) who suspected ARFID misapplied the exclusion criteria, resulting in misdiagnosis.

In contrast, Claudino et al. 2019 used vignettes to assess HCPs proficiency in utilising the new ICD‐11 guidelines. This international investigation revealed that the ICD‐11 guidelines were favourably rated over ICD‐10 for each feeding and eating disorder diagnosis in terms of ease of use, clarity and alignment with the presented vignettes. Under the ICD‐11 guidelines, HCPs demonstrated a higher ability to distinguish between ARFID cases (88.5% correct) and those without a diagnosis (78.4% correct). The authors concluded that the inclusion of ARFID in the ICD‐11, simplified the diagnostic process and HCPs utilising these guidelines were better equipped to distinguish ARFID cases from anorexia.

### 3.3 Need for Clear Referral Pathways and Enhancing Multidisciplinary Collaboration

#### 3.3.1 Pathways and Referrals

Harrison 2021 found 67% of HCPs expressed uncertainty about where to refer children for an ARFID assessment and less than half of both primary (28%) and secondary (41%) care HCPs felt they knew where to make onward referrals for their treatment and support. This lack of clarity poses considerable challenges for families seeking appropriate identification and timely referrals to specialist services. For example, Dinkler et al. 2023 found a significant majority (89.3%) of Swedish HCPs expressed uncertainty about how to approach ARFID, with only 15.5% of HCPs specialising in eating disorders and 14.3% of Physicians and Psychologists reporting knowing how to treat ARFID. Jackson et al. 2022 further noted the absence of clear referral criteria and professional support once children are deemed medically stable, exacerbating the challenge of appropriate referrals. HCPs even suggested some services had purposely exclusionary referral criteria, with children with symptoms of Autism being particularly likely to be rejected on this basis (Harisson 2025).

Magel et al. 2021 revealed a trend among Canadian community HCPs having to provide treatment for ARFID without any training (57.1%). This shortfall in expertise was compounded by a lack of guidance on where to refer patients for specialised care, forcing many HCPs to navigate ARFID treatment independently, with varying degrees of success with a wide arrange of treatment methods employed, including food exposure, hypnotherapy, nutritional counselling and integrative psychotherapy.

Guss et al. 2018 completed a survey of US Physician practices concerning the inpatient medical stabilisation of patients with ARFID, and found even with standardised protocols in place, more than half used the same protocol for ARFID as they did for anorexia. A mere 22.7% of the sample reported having a non‐anorexia refeeding protocol specifically designed for ARFID patients, highlighting a notable gap.

#### 3.3.2 Lack of Multi‐Disciplinary Team (MDT) Working

Despite recognising the value of MDTs, many New Zealander and British HCPs tended to refer to single professions due to the lack of suitable services, particularly in smaller communities where access to multidisciplinary teams is limited (Coelho et al. 2021; Jackson et al. 2022). Reliance on individual clinician judgement in making a diagnosis often exacerbated HCPs’ lack of confidence (26).

The challenges of limited access to MDTs were compounded by the reluctance of services to accept referrals for ARFID patients, as noted by Harrison 2021. Many relevant services, including Psychologists, Dietetics, Speech and Language and Specialist Nursing teams, acknowledged the need for a multidisciplinary approach but often declined referrals as this approach was lacking. This situation leaves patients with ARFID in a precarious position. Guss et al. 2018 observed that many United States Physicians caring for inpatient ARFID cases were part of MDTs and the necessity of MDTs in its management.

## 4 Discussion

The current review revealed a pervasive gap in HCPs’ awareness and confidence in understanding ARFID (Harrison 2021; Dinkler et al. 2023; Jackson et al. 2022), with significant challenges that HCPs face in identifying and diagnosing ARFID (Katzman et al. 2014; Magel et al. 2021; Harrison 2021; Coelho et al. 2021; Dinkler et al. 2023). This was compounded by differing diagnostic manuals and a lack of validated assessment tools, thus strengthening the reliance on clinical judgement despite lack of confidence. Furthermore, this review highlights the lack of specific training and awareness among HCPs regarding the nuances of ARFID, particularly in the context of conditions where there is an overlap in symptom presentation, such as normative picky eating and Autism.

Highlighting the ethical dilemma faced by HCPs, Magel et al. 2021draws attention to the tension between the ethical obligation to provide care and the imperative to balance the lack of training and remaining within the bounds of clinical competence. Furthermore, many HCPs expressed a lack of awareness regarding how to acquire the necessary skills to effectively treat ARFID, a predicament compounded its novelty and the limited availability of resources. Indeed, Tan and Johns 2021study involving NHS trusts across England and Scotland, found as many as 62% of the eating disorders services surveyed were unable to modify treatment for eating disorders for patients with co‐occurring conditions due to a lack of training. This lack of preparedness may contribute to misdiagnoses and/or delayed diagnoses.

Despite the evolving and developing landscape of ARFID recognition and service provision, findings from some of the more recent studies suggest that there remain persistent gaps in identification, training and service pathways. Harrison (26) found that both primary and specialist HCPs reported difficulties in identifying ARFID and uncertainty regarding referral pathways, with limited knowledge, training and availability of clear services identified as key barriers to effective diagnosis and treatment.

The lack of clear referral pathways and multidisciplinary collaboration were also raised as significant barriers in the diagnosis and treatment of ARFID (Jackson et al. 2022). For example, HCPs report confusion about the availability of services and referral processes, compounded by stringent specialist service exclusion criteria and commissioning gaps leaving patients without any structured support (Coelho et al. 2021). This situation calls for a more integrated and informed approach to diagnosing ARFID, one that acknowledges the complex interplay of co‐occurring conditions and values the insights of caregivers.

Although many studies highlighted gaps in service provision, some healthcare systems have begun implementing more structured pathways for ARFID care. For example, in 2019 Australia introduced the Medicare‐Supported Pathway through new Medicare Benefits Schedule (MBS) items for all eating disorders, inclusive of Avoidant/Restrictive Food Intake Disorder (McLean and Fuller‐Tyszkiewicz 2024). This model requires general practitioners to develop an Eating Disorder Treatment and Management Plan (EDP) and facilitates access to a multidisciplinary team, typically involving psychologists and dietitians. Under this pathway, eligible individuals can receive up to 40 Medicare‐subsidised psychological treatment sessions and up to 20 dietetic sessions within a 12‐month period, reflecting a stepped‐care, evidence‐based approach to treatment intensification according to clinical need.

### 4.1 Limitations

While this review is the first to synthesise the experiences of HCPs working with individuals with ARFID, several limitations should be acknowledged. First, the review did not incorporate the perspectives of service users and their families regarding their interactions with HCPs. This omission is partly due to the limited availability of literature exploring how service users and families experience encounters. As a result, this review does not fully capture how HCP practices are perceived or how they influence the overall help‐seeking process and care journey. Further research is needed to explore these perspectives and their role in shaping access to, engagement with and outcomes of care.

Additionally, most included studies focused on children and adolescents, which may limit the transferability of findings to adult ARFID presentations and adult service contexts. The heterogeneity of the HCPs, service settings and international healthcare systems represented across studies may also limit the generalisability and practical applicability of findings across disciplines and geographical contexts.

Furthermore, the HCPs with greater experience or interest in ARFID may have been more likely to participate in the included studies, potentially contributing to sampling bias. This possibility is reflected in the overrepresentation of ARFID interventions originating from specialist eating disorder services and settings (Sanchez‐Cerezo et al. 2023).

Methodological considerations should also be acknowledged. We chose to focus on clear, current definitions of ARFID rather than older studies, as retrospectively assigning ARFID diagnoses to Paediatric Feeding Disorder‐labelled studies would involve subjective interpretation of inadequately reported symptoms, exclusion criteria and levels of impairment. However, this approach may limit the generalisability of the findings by relying on clearly defined presentations while underrepresenting the broader or more complex feeding difficulties seen in clinical practice.

### 4.2 Implications for Practice

Eating disorders such as AFRID are recognised as having high levels of co‐occurrence with other diagnoses and require management by sufficiently skilled HCPs (Tan and Johns 2021). The findings of this review highlight the ongoing need for formalised training programs and resources tailored specifically to ARFID, to ensure that HCPs are equipped with the knowledge and skills required to confidently identify and manage this heterogenous disorder across the lifespan and clinical settings.

However, the implications of these findings are unlikely to be uniform across professional groups. The knowledge, responsibilities and scope of practice relating to ARFID differ considerably across disciplines. As such, training and service development initiatives should be tailored to the specific roles and clinical responsibilities of different professional groups, rather than assuming a universal approach to ARFID assessment and management.

Moreover, the findings also highlight the importance of multidisciplinary collaboration in ARFID care. Difficulties accessing multidisciplinary teams, reliance on individual clinician judgement and inconsistent referral pathways across service (Harrison 2021; Coelho et al. 2021; Jackson et al. 2022) suggest that reliance on a single‐service or single‐discipline framework may be insufficient to address the complexity of presentations commonly encountered in practice. While ARFID is often conceptualised within eating disorder services, this positioning may inadvertently overlook the expertise of multidisciplinary professionals, including paediatricians, dietitians, speech and language therapist, occupational therapists and neurodevelopmental services, who may play an important role in assessment and intervention (Duffy et al. 2024). This is further supported by emerging literature emphasising the benefit of multidisciplinary approaches in ARFID and paediatric feeding care (Perez and Pesce 2025).

## 5 Conclusions

Overall, this review highlights the need for stronger partnerships between research and clinical communities, including paediatric feeding specialists, to move beyond the traditional eating disorder lens when understanding and managing ARFID. While included studies reflect an evolving and developing landscape of ARFID recognition and service provision, persistent gaps remain in multidisciplinary collaboration, HCP training and referral pathways. Addressing these barriers may support improvements in diagnostic accuracy and treatment outcomes, particularly for children and adolescents with ARFID. Future research should further explore how emerging models of multidisciplinary care can be effectively implemented across different healthcare settings and countries to support accessible, comprehensive and developmentally appropriate care.

## Funding

The authors have nothing to report.

## Ethics Statement

The authors have nothing to report.

## Conflicts of Interest

The authors declare no conflicts of interest.

## Data Availability

The data that support the findings of this study are available from the corresponding author upon reasonable request.
